# Sport-based psychosocial interventions for people suffering from severe mental disorders: EASMH pilot actions from 4 European Countries

**DOI:** 10.1192/j.eurpsy.2024.160

**Published:** 2024-08-27

**Authors:** M. Di Vincenzo, G. Sampogna, M. Borgi, B. Collacchi, F. Cirulli, S. Cerino, S. Rullo, M. Luciano, V. Di Tommaso, S. Moliterni, A. Bichi, J. Garside, S. Kivistö, A. Iarion, M. Walker, A. Fiorillo

**Affiliations:** ^1^Department of Psychiatry, University of Campania “Luigi Vanvitelli”, Naples; ^2^Center for Behavioral Sciences and Mental Health, National Institute of Health; ^3^European Culture and Sport Organization, Rome, Italy; ^4^The European Platform for Sport Innovation, Brussels, Belgium; ^5^Everton in the Community Ltd, Liverpool, United Kingdom; ^6^Finnish Sport Federation, Tempere, Finland; ^7^Faculty of Physical Education and Sport, University of Costanta, Costanta, Romania; ^8^European Psychiatric Association, Strasbourg, France

## Abstract

**Introduction:**

The *European Alliance for Sport and Mental Health* (EASMH) is a partnership of scientific institutions, charity associations and sport organizations, funded by EU-Erasmus+. It aimed at developing good clinical practice in psychiatric rehabilitation through sport-based interventions as an integration of pharmacological and psychological therapies. Within the framework of the EASMH projects, several actions have been promoted including an assessment of the dissemination of sport-based interventions, a training course for specialized coaches and the implementation of pilot actions in four European Countries.

**Objectives:**

To briefly describe EASMH pilot actions performed in Finland, Italy, Romania and United Kingdom, where trained coaches delivered sport-based interventions to patients with severe mental disorders.

**Methods:**

After completing pilot actions, charity associations and sport organizations belonging to EASMH network described general and specific aims, sport activities, composition of staff, timing and tools for assessing the outcomes.

**Results:**

In Italy, “Crazy for Rugby”, including adolescents and young patients, and “Not only headshots”, a football project for adults with severe mental disorders were performed. In UK, a football-based activity called “Imagine Your Goal” and a walking-football program for participants aged more than 40 were delivered. In Romania, two courses including gymnastics, yoga and pilates called “Get fit!” were provided. Different team sport-based activities were implemented in Finland, where “Multiple Sport Group” and “Rehabilitating Sports” aimed at increasing patients’ autonomy. Assessment of psychopathological, social, cognitive and sport/fitness outcomes confirmed the overall beneficial effects of sport on mental health.

**Conclusions:**

Pilot actions represent the final step of EASMH project, which showed improvement of mental health outcomes by also delivering sport-based rehabilitation to patients with severe mental disorders. Institutions and stakeholders are now called to promote the implementation of such initiatives on a broader scale.

**Disclosure of Interest:**

None Declared

